# Bisphenol A exposure in myasthenia gravis: Potential targets and mechanisms revealed by network toxicology and molecular dynamics

**DOI:** 10.1371/journal.pone.0354138

**Published:** 2026-07-28

**Authors:** Shupeng Wu, Yaoqi Wu, Li Zhang, Jun Zhu

**Affiliations:** 1 Zhengzhou Hospital of Traditional Chinese Medicine Affiliated to Henan University of Chinese Medicine, China; 2 Science and Technology Innovation Center, Guangzhou University of Chinese Medicine, Guangzhou, China; 3 The First School of Clinical Medicine, Henan University of Chinese Medicine, Zhengzhou, China; University of Hyderabad, INDIA

## Abstract

**Background:**

Myasthenia gravis (MG) is a B-cell-mediated autoimmune disease characterized by impaired neuromuscular transmission. Although genetic predisposition and thymic abnormalities are well recognized, they cannot fully explain the increasing incidence and regional heterogeneity of MG, highlighting the potential contribution of environmental factors. Bisphenol A (BPA), a ubiquitous endocrine-disrupting chemical, exhibits estrogenic activity, immunomodulatory effects, and mitochondrial toxicity, and has been implicated in multiple autoimmune disorders. However, the potential role of BPA in MG pathogenesis remains largely unexplored.

**Methods:**

BPA-related targets and MG-associated genes were collected from public databases, and overlapping targets were identified. Gene Ontology (GO) and Kyoto Encyclopedia of Genes and Genomes (KEGG) enrichment analyses were performed to explore the biological functions and pathways of the overlapping targets. Candidate targets were screened using four machine-learning algorithms, including least absolute shrinkage and selection operator (LASSO) regression, support vector machine-recursive feature elimination (SVM-RFE), random forest (RF), and extreme gradient boosting (XGBoost). Differential expression and diagnostic performance were validated using the Gene Expression Omnibus (GEO) dataset GSE85452. Immune infiltration was assessed using CIBERSORT. Molecular docking and molecular dynamics (MD) simulations were conducted to evaluate the potential binding stability and interaction modes between BPA and key target proteins. In addition, C2C12 myoblasts were treated with different concentrations of BPA for 24 h, and cell viability was assessed using the Cell Counting Kit-8 (CCK-8) assay. Based on the cell viability results, 50 μM BPA was selected for quantitative reverse transcription polymerase chain reaction (qRT-PCR) and Western blot analyses of key genes.

**Results:**

A total of 225 overlapping targets were identified between BPA exposure-related targets and MG-associated genes. Enrichment analyses showed that these targets were mainly associated with ion transport, membrane potential regulation, muscle system processes, immune signaling, apoptosis, endocrine resistance, and AGE–RAGE signaling pathways.Four machine-learning algorithms identified cholinergic receptor nicotinic beta 1 subunit (CHRNB1), KRAS proto-oncogene, GTPase (KRAS), phosphomannomutase 2 (PMM2), and toll-like receptor 4 (TLR4) as candidate targets. Validation in the GSE85452 dataset showed that CHRNB1, PMM2, and TLR4 were significantly upregulated in MG samples compared with control samples, whereas KRAS showed no significant difference. receiver operating characteristic (ROC) analysis further demonstrated that CHRNB1, PMM2, and TLR4 had good diagnostic performance, with area under the ROC curve (AUC) values of 0.827, 0.856, and 0.821,respectively. Immune infiltration analysis revealed altered immune cell infiltration patterns and associations between key targets and specific immune cell subsets. Molecular docking predicted favorable binding of BPA to CHRNB1, PMM2, and TLR4, with binding energies of −7.4, −5.7, and −5.8 kcal/mol, respectively. MD simulations further supported the potential stability of these BPA-target complexes. In vitro experiments showed that BPA reduced C2C12 cell viability in a concentration-dependent manner. qRT-PCR validation showed that treatment with 50 μM BPA significantly upregulated Chrnb1 expression, while downregulating Pmm2 and Tlr4 expression.Western blot analysis further confirmed that BPA exposure significantly decreased the protein expression of TLR4 and PMM2, while increasing that of CHRNB1 in C2C12 cells.

**Conclusions:**

This study provides integrated computational and experimental evidence that BPA exposure may be associated with MG-related molecular alterations. BPA may affect MG-related biological processes through the regulation of ion transport, neuromuscular signaling, immune activation, and glycosylation-related metabolism. CHRNB1, PMM2, and TLR4 may serve as potential molecular links between BPA exposure and MG-related pathological processes.

## 1 Introduction

Myasthenia gravis (MG) is a chronic autoimmune neuromuscular disorder characterized by dysfunction of neuromuscular transmission, which manifests clinically as fluctuating skeletal muscle weakness and fatigability [1]. Epidemiological studies indicate that the incidence of MG spans 1.7–21.3 cases per million person-years, while the global average incidence is estimated at 5.3 cases per million person-years [2]. This disease is a B-cell-mediated autoimmune disorder characterized by autoantibody production. These autoantibodies mainly target key components of the neuromuscular junction, including the acetylcholine receptor (AChR), muscle-specific kinase, and lipoprotein receptor-related protein 4 (LRP4) [3,4]. These antibody-mediated attacks disrupt postsynaptic signal reception and ultimately lead to impaired neuromuscular transmission [5]. Among patients with anti-AChR antibodies, immune regulatory dysfunction, thymic abnormalities, and hormonal factors are recognized as key contributors to disease pathogenesis. Although therapeutic strategies including immunosuppressive agents and cholinesterase inhibitors have advanced, the exact etiology of MG remains incompletely understood [6]. Growing attention has been directed toward environmental factors, particularly endocrine-disrupting chemicals (EDCs) [7].

Bisphenol A (BPA), a typical EDC widely found in plastic products, food packaging, and medical devices, has been demonstrated to possess estrogen-like activity, immunomodulatory potential, and mitochondrial toxicity [8,9]. Human exposure to BPA is widespread worldwide, mainly through dietary intake and migration from food-contact materials, and urinary BPA is commonly used as a biomarker of internal exposure because BPA is rapidly metabolized and excreted in urine [10–12]. NHANES and global biomonitoring studies have shown widespread BPA exposure with regional and population-specific variability [13,14], while recent EFSA risk assessment reported that dietary exposure estimates exceeded the updated tolerable daily intake of 0.2 ng/kg bw/day across all age groups [15].Epidemiological and experimental evidence indicates that BPA exposure is linked to heightened susceptibility to autoimmune conditions, including systemic lupus erythematosus and rheumatoid arthritis [16,17]. However, the potential link between BPA and MG has not yet been systematically investigated. Due to the essential role of immune homeostasis disruption and neuromuscular junction microenvironment dysregulation in MG pathogenesis, whether BPA contributes to the development or progression of MG by interfering with the imm une system, affecting the neuroendocrine axis, or directly impairing muscle function represents a critical scientific question.

Traditional toxicological studies have often focused on single targets or pathways, making it difficult to comprehensively elucidate the effects of a pleiotropic compound like BPA in complex diseases. In recent years, computational biology approaches integrating network toxicology and molecular docking have provided powerful tools for systematically unraveling the potential toxic mechanisms of chemical exposure [18]. Network toxicology is a core discipline that drives the transformation of the research paradigm in toxicology. From a system-level perspective, it connects chemical exposure with toxic outcomes, accelerating the discovery of toxic mechanisms and the prediction of safety risks, and demonstrating strong application value in the fields of traditional Chinese medicine, environmental health, and drug safety [19–21]. Building on these predictions, molecular docking and MD simulation were used to explore compound-protein interactions at the atomic level, evaluating binding modes and affinities to validate and refine the proposed mechanisms.

This study adopts an integrative framework combining network toxicology, machine learning, molecular docking, and MD simulation to systematically identify potential molecular targets and pathways through which BPA exposure may influence MG. We further explore the biological mechanisms by which BPA might participate in MG pathology by interfering with immune regulation, neural signaling, or energy metabolism. The findings are expected to offer novel theoretical perspectives on the role of environmental factors in MG pathogenesis and offer potential molecular clues for risk assessment and intervention strategies.

## 2 Materials and methods

### 2.1 Identification of BPA-related targets

**Fig 1:**
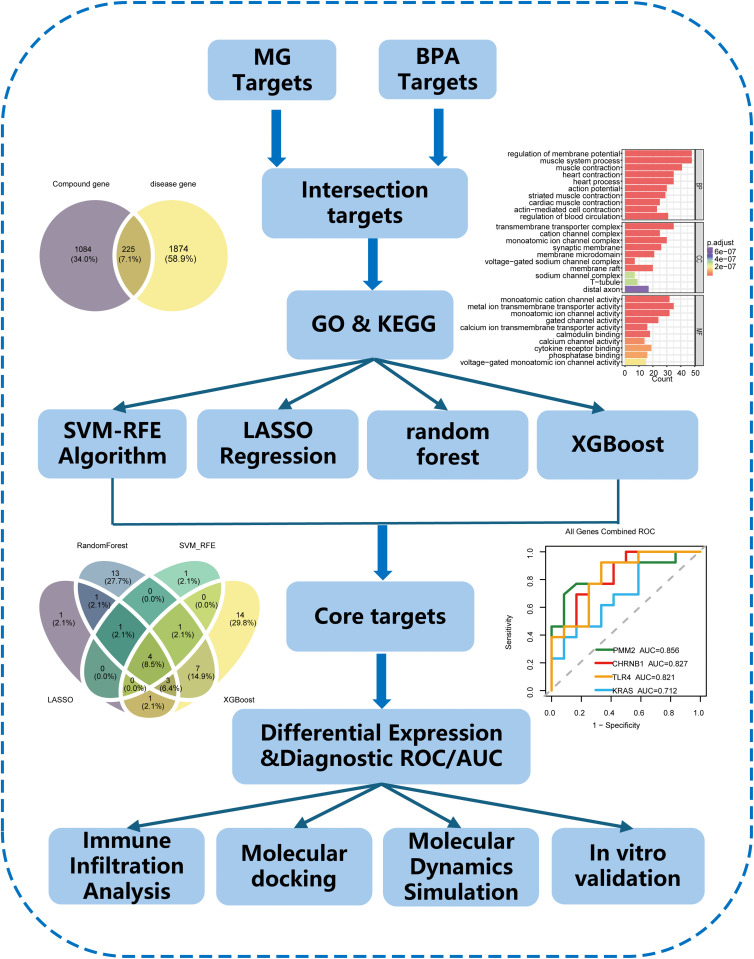
Analysis of the Workflow for Toxicological Mechanisms of BPA in MG.

The design methodology and flow of this study is demonstrated in Fig 1. BPA action targets were collected as follows. The chemical structure and SMILES representation of BPA were obtained from the PubChem database (https://pubchem.ncbi.nlm.nih.gov/). Potential BPA action targets were then screened with the species limited to “Homo sapiens” using the ChEMBL (https://www.ebi.ac.uk/chembl/), STITCH (http://stitch.embl.de/), and Swiss Target Prediction (http://swisstargetprediction.ch/) databases [22]. A broad target collection strategy was adopted to comprehensively identify potential BPA-associated targets; therefore, no additional confidence-score or probability-score cutoff was applied during the initial retrieval. All retrieved targets were standardized to official gene symbols, and non-human entries, duplicate records, ambiguous targets, obsolete gene symbols, and targets without valid gene annotations were removed. When confidence scores, interaction scores, or prediction probabilities were available from the original databases, they were retained for reference but were not used as exclusion criteria. Finally, the curated targets from the three databases were integrated and deduplicated to generate a nonredundant BPA-related target library.

### 2.2 Acquisition of potential MG-related targets

Disease-related targets for MG were obtained from the OMIM (https://omim.org/), DisGeNET (https://disgenet.com/), and GeneCards (https://www.genecards.org/) databases using “myasthenia gravis” as the search term. To comprehensively collect potential MG-related genes, no additional score-based cutoff was applied during the initial retrieval. All retrieved targets were standardized to official gene symbols, and non-human entries, duplicate records, ambiguous genes, obsolete gene symbols, and genes without valid annotations were excluded. For databases that provided relevance scores or gene-disease association scores, such as GeneCards and DisGeNET, these scores were recorded for reference but were not used as exclusion criteria. The curated targets from the three databases were then merged and deduplicated to obtain the final set of potential MG-related targets.

### 2.3 Functional enrichment analysis of target genes

Functional enrichment analyses were conducted to explore the biological roles of BPA-induced MG target genes. Gene Ontology (GO) terms, including Biological Process (BP), Cellular Component (CC), and Molecular Function (MF), as well as Kyoto Encyclopedia of Genes and Genomes (KEGG) signaling pathways, were analyzed using the DAVID platform (https://david.ncifcrf.gov/). Enriched functional categories and pathways were determined based on an adjusted *P* < 0.05.

### 2.4 Identification of core targets using machine learning approaches

Four machine-learning approaches, including least absolute shrinkage and selection operator (LASSO) regression, support vector machine-recursive feature elimination (SVM-RFE), random forest (RF), and extreme gradient boosting (XGBoost), were applied to identify core targets related to BPA-associated MG. The intersecting genes between BPA-related targets and MG-related targets were used as candidate variables, and MG/control status was used as the binary outcome variable.LASSO regression was performed using the glmnet R package. A binomial model was constructed with family = “binomial” and alpha = 1. The random seed was set to 12345. Ten-fold cross-validation was performed using the cv.glmnet function, with deviance as the evaluation criterion. The optimal penalty parameter was selected according to the minimum cross-validation error (lambda.min), and genes with nonzero coefficients were retained as LASSO-selected features.SVM-RFE was performed using the e1071 R package. The gene expression matrix was standardized before model training. A linear-kernel SVM classifier was used for recursive feature ranking, with the cost parameter set to 10. Ten-fold cross-validation was applied to evaluate feature subsets, and the optimal number of features was determined according to the lowest mean cross-validated error rate. During model validation, SVM parameters were optimized by grid search.Random forest analysis was performed using the randomForest R package. A classification model was constructed with 500 decision trees, and the number of variables randomly sampled at each split was set to the square root of the total number of input genes. Feature importance was evaluated using the MeanDecreaseGini index, and the top 30 genes ranked by MeanDecreaseGini were retained as RF-selected features.XGBoost analysis was performed using the xgboost R package. A binary logistic classification model was constructed with the objective function set as binary:logistic and the evaluation metric set as area under thereceiver operating characteristic curve (AUC). The main parameters included max_depth = 3, eta = 0.05, subsample = 0.8, colsample_bytree = 0.8, and nrounds = 100. Feature importance was calculated using the built-in importance function, and the top 30 genes ranked by XGBoost importance score were retained as XGBoost-selected features.The final core targets were defined as the overlapping genes identified by the four machine-learning algorithms.

### 2.5 Evaluation of machine-learning model performance and diagnostic value

To compare the classification performance of the four machine-learning models, ten-fold cross-validation was performed for LASSO, SVM-RFE, RF, and XGBoost. For each model, the predicted probability of each sample was obtained, and model performance was evaluated using accuracy, sensitivity, specificity, precision, F1 score, and AUC. Receiver operating characteristic (ROC) curves were generated using the pROC R package, and the AUC values were calculated to assess the discriminative ability of each model.For the final core targets, ROC analysis was further performed to evaluate their diagnostic value in distinguishing MG samples from control samples. The ROC curve was plotted using sensitivity against 1-specificity, and the AUC with 95% confidence interval was calculated. Genes with significant differential expression and favorable AUC values were considered potential diagnostic biomarkers.

### 2.6 Core gene expression profiling and diagnostic performance evaluation

Transcriptomic data from MG and healthy control peripheral blood-derived CD14 + monocyte samples were obtained from the Gene Expression Omnibus (GEO) dataset GSE85452. The expression matrix of the screened core genes was extracted for subsequent analysis. Sample groups were defined according to the sample labels, with healthy control samples coded as 0 and MG samples coded as 1. The expression levels of the core genes were compared between MG and control samples using Student’s t-test, and genes with *P* < 0.05 were considered significantly differentially expressed. Boxplots and density plots were generated to visualize the expression distribution of each core gene between the two groups.ROC curve analysis was performed using the pROC R package to evaluate the diagnostic relevance of the screened core genes. The AUC and corresponding 95% confidence interval were calculated for each gene. The optimal cutoff value was determined using the Youden index, and sensitivity, specificity, and accuracy were calculated according to the corresponding cutoff. Individual ROC curves were generated for each core gene, and all ROC curves were further integrated into a combined plot for comparison.

### 2.7 Immune infiltration analysis

Immune infiltration analysis was performed using a CIBERSORT-based deconvolution approach to estimate the relative proportions of immune cell types in CD14 + monocyte samples from MG patients and healthy controls [23]. The expression matrix was used as the mixture file, and a reference immune cell signature matrix was used as the reference file. The CIBERSORT algorithm was implemented using support vector regression with quantile normalization. The number of permutations was set to 1,000, and samples with CIBERSORT output *P* < 0.05 were retained for subsequent analysis. Differences in immune cell infiltration between MG and control samples were evaluated using the Wilcoxon rank-sum test. Associations between immune infiltration patterns and core target gene expression were further analyzed [24].

### 2.8 Molecular docking

Molecular docking was performed to evaluate the binding affinity between BPA and the key target proteins. The experimentally resolved protein structures ofcholinergic receptor nicotinic beta 1 subunit (CHRNB1), phosphomannomutase 2 (PMM2), and toll-like receptor 4 (TLR4) were preferentially obtained from the RCSB Protein Data Bank when available, and the corresponding PDB IDs were 9GU0, 7O0C, and 5NAM, respectively. For targets without suitable experimentally resolved structures, the predicted structures were obtained from the AlphaFold Protein Structure Database using the corresponding UniProt accession numbers. The three-dimensional structure of BPA was obtained from the PubChem database and was geometrically optimized before docking.Before docking, all protein structures were preprocessed using PyMOL and AutoDockTools. Crystallographic water molecules, co-crystallized ligands, and irrelevant heteroatoms were removed. Missing atoms or residues were checked and repaired when necessary. Polar hydrogen atoms were added, and atomic charges were assigned. Energy minimization was then performed to remove unfavorable steric clashes and refine the local geometry of the protein structures. The prepared receptor and ligand files were converted into PDBQT format for molecular docking.Molecular docking was performed using AutoDock Vina. The binding energy was used to evaluate the binding affinity between BPA and the target proteins. The docking poses with the lowest binding energy were selected for subsequent visualization and MD simulations [25].

### 2.9 MD simulation

Molecular dynamics simulations were conducted using GROMACS 2024.4 to study the interactions betweenBPA and the target proteins CHRNB1, PMM2, and TLR4. The AMBER14SB and GAFF2 force fields were used for protein and ligand parameterization, respectively, and the TIP4P water model was used to solvate each system in a periodic box with a 1.2 nm buffer. Long-range electrostatic interactions were calculated using the PME method, and sodium and chloride ions were added by Monte Carlo ion placement to neutralize the system charge. Energy minimization and equilibration were performed in three steps: (1) 50,000 steps of steepest descent energy minimization until the maximum force was < 1000 kJ/mol, (2) NVT pre-equilibration at 310 K for 50,000 steps, and (3) NPT pre-equilibration at 310 K for 50,000 steps. After equilibration, production simulations were run for 100 ns with a 2 fs time step, and coordinates were saved every 10 ps. Trajectory analyses were performed using root-mean-square deviation (RMSD), root-mean-square fluctuation (RMSF), radius of gyration (Rg), solvent-accessible surface area (SASA), protein–ligand hydrogen bonds, free energy distributions, and time-point structural comparisons at 0, 25, 50, 75, and 100 ns.To further evaluate the thermodynamic stability of the BPA-target protein complexes, molecular mechanics Poisson–Boltzmann surface area (MM-PBSA) binding free energy analysis was performed based on the equilibrated MD trajectories.. The binding free energy was calculated as follows:


$$\begin{array}{c} \Delta G_{bind}=G_{complex}-G_{protein}-G_{ligand} \end{array}$$

where ΔG_bind_ represents the binding free energy of the protein–ligand complex. The total binding free energy was decomposed into molecular mechanics energy, polar solvation energy, nonpolar solvation energy, and entropy contribution. A more negative ΔG_bind_ value was considered to indicate a more favorable and stable interaction between BPA and the target protein.

### 2.10 Quantitative reverse transcription polymerase chain reaction validation

Mouse C2C12 myoblasts were purchased from the Cell Bank of the Chinese Academy of Sciences. Cells were maintained under standard culture conditions and assigned to either the Control group or the BPA-treated group. The mouse orthologs of the selected human key target genes were used for quantitative reverse transcription polymerase chain reaction (qRT-PCR) validation in C2C12 cells.To determine a suitable BPA concentration for subsequent experiments, C2C12 cells were exposed to BPA at concentrations of 1, 10, 50, and 100 μM for 24 h, after which cell viability was assessed. According to the results of the cell viability assay, 50 μM BPA was selected for qRT-PCR validation. For this experiment, cells in the BPA-treated group were incubated with 50 μM BPA for 24 h, whereas cells in the Control group were cultured under the same conditions without BPA exposure.Following treatment, total RNA was isolated from C2C12 cells using the TRIzol method in accordance with the manufacturer’s protocol. RNA concentration and purity were determined using a NanoDrop spectrophotometer. Subsequently, 1 μg of total RNA was reverse-transcribed into cDNA using a reverse transcription kit. Quantitative real-time PCR was then carried out on an ABI 7500 Real-Time PCR System using SYBR Green Master Mix.The relative expression levels of target genes were normalized to β-Actin as the internal reference gene and calculated using the 2 − ΔΔCt method. All qRT-PCR experiments were performed with at least three independent biological replicates. The primer sequences used for qRT-PCR are listed in Table 1.

**Table 1:** Primer sequences used for real-time qRT-PCR.

Gene	Species	Orientation	Primer sequences(5’–3’)	Product size (bp)
*Chrnb1*	*Mus musculus*	Forward Reverse	AGGAGAGGAGAGGCAGGAAG CACCTCTCTGATCCCCTGGA	116
*Pmm2*	*Mus musculus*	Forward Reverse	GAAAGGCCTCACGTTCTCCA TCTCATGGTCATTGCCACCC	158
*Tlr4*	*Mus musculus*	Forward Reverse	CTGGGGAGGCACATCTTCTG CCTCTGCTGTTTGCTCAGGA	82

### 2.11 Western blot analysis

To further validate the changes in the protein expression levels of the selected candidate genes, Western blot analysis was performed using C2C12 cells. According to the results of the cell viability assay, cells in the BPA-treated group were exposed to 50 μM BPA for 24 h, while cells in the Control group were cultured under the same conditions without BPA treatment.After treatment, C2C12 cells were washed three times with cold phosphate-buffered saline (PBS) and lysed with RIPA lysis buffer containing protease inhibitor. The cell lysates were collected and centrifuged at 12,000 rpm for 15 min at 4 °C, and the supernatants were obtained for total protein extraction. Protein concentrations were determined using a BCA protein assay kit according to the manufacturer’s instructions. Equal amounts of protein samples were separated by SDS-PAGE and subsequently transferred onto PVDF membranes.

The membranes were blocked with 5% non-fat milk at room temperature for 1 h and then incubated overnight at 4 °C with the following primary antibodies: CHRNB1 polyclonal antibody (cat. no. 11553–1-AP, Proteintech Group, Inc., Rosemont, IL, USA; 1:2000), anti-PMM2 rabbit polyclonal antibody (cat. no. P105825, Shanghai Epizyme Biomedical Technology Co., Ltd., Shanghai, China; 1:2000), Toll-like receptor 4 (TLR4) polyclonal antibody (cat. no. PAA753Mu01, Cloud-Clone Corp., Wuhan, China; 1:2000), anti-GAPDH rabbit monoclonal antibody (cat. no. PTM-5375, PTMab, Hangzhou, China; 1:5000), and anti-β-Actin mouse monoclonal antibody (cat. no. PTM-5018, PTMab, Hangzhou, China; 1:2000). GAPDH and β-Actin were used as internal loading controls. After washing with TBST, the membranes were incubated with Multi-rAb® HRP-Goat Anti-Rabbit Recombinant Secondary Antibody (cat. no. RGAR001, Proteintech Group, Inc. / Wuhan Sanying Biotechnology Co., Ltd., Wuhan, China; 1:6000) and Multi-rAb® HRP-Goat Anti-Mouse Recombinant Secondary Antibody(cat. no. RGAM001, Proteintech Group, Inc. / Wuhan Sanying Biotechnology Co., Ltd., Wuhan, China; 1:6000) at room temperature for 1 h.Finally, the protein bands were visualized using an enhanced chemiluminescence detection system. The gray values of the bands were quantified using ImageJ software, and the relative protein expression levels of target proteins were normalized to their corresponding internal loading controls. All experiments were performed with at least three independent biological replicates.

### 2.12 Statistical analysis

Statistical analyses were performed using R software (version 4.5.1) and GraphPad Prism. Continuous variables are presented as the mean ± standard deviation, whereas categorical variables are reported as frequencies and percentages. Comparisons of mRNA expression levels between the Control and BPA-treated groups were performed using Student’s t-test. A value of *P* < 0.05 was considered statistically significant.For bioinformatics analyses, functional enrichment results were considered statistically significant based on an adjusted *P* < 0.05. Differential expression analysis was performed using Student’s t-test, and genes with *P* < 0.05 were considered significantly differentially expressed. For immune infiltration analysis, statistical differences between groups were examined using the Wilcoxon rank-sum test.To evaluate the diagnostic accuracy of the candidate genes, ROC curves were generated using the pROC R package. The AUC and corresponding 95% confidence interval were calculated. The optimal cutoff value was determined using the Youden index, and the corresponding sensitivity, specificity, and accuracy were calculated. ROC curves were constructed by plotting sensitivity against 1-specificity. All statistical tests were two-sided unless otherwise stated.

## 3 Results

### 3.1 Network toxicology analysis of candidate targets linked to BPA induced MG

**Fig 2:**
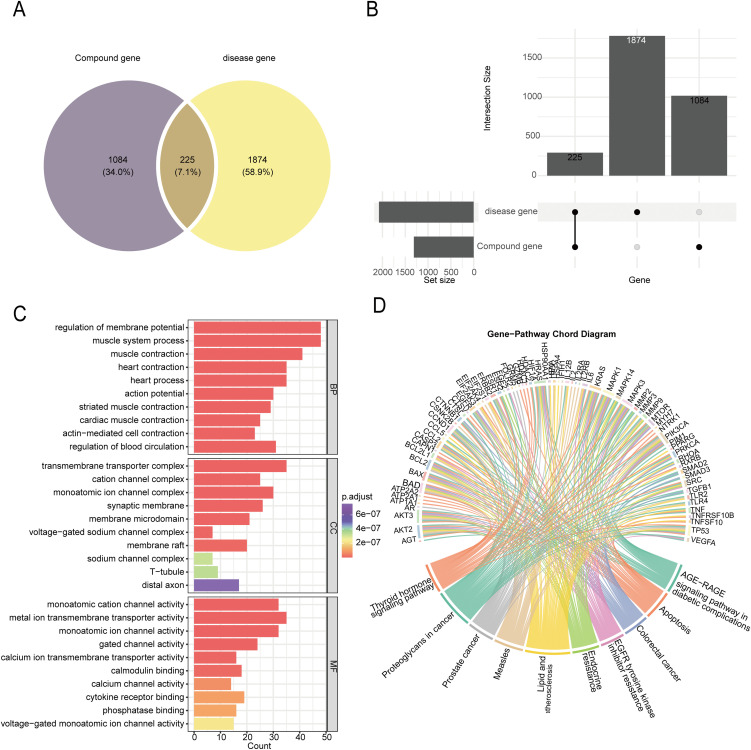
Network toxicology analysis of candidate targets linked to BPA induced MG. A. Venn diagram of BPA-associated targets and MG-related genes. B. Upset diagram of BPA- related targets and MG-associated genes. C. GO pathway enrichment analysis of BPA-induced MG targets. D. KEGG pathway enrichment analysis of BPA-induced MG targets.

The potential targets of BPA-induced MG were systematically investigated through network toxicology. In total, 1309 potential BPA-associated targets were collected from the ChEMBL, STITCH, and Swiss Target Prediction databases. Meanwhile, 2099 MG-related targets were collected from the OMIM, DISGENET and GeneCards databases. Integration of the two target sets resulted in 225 shared targets, highlighting key candidates potentially involved in BPA-induced MG (Fig 2A-B).Furthermore, GO and KEGG enrichment analyses were applied to explore functional enrichment among the overlapping targets (Fig 2C-D). The GO analysis indicated that these targets were significantly enriched in biological processes such as regulation of membrane potential and muscle system process, cellular components including transmembrane transporter complex and cation channel complex, and molecular functions like monoatomic cation channel activity and metal ion transmembrane transporter activity. KEGG pathway analysis highlighted significant involvement in Lipid and atherosclerosis, Apoptosis, Endocrine Resistance, and AGE-RAGE signaling pathway, which are known to be associated with autoimmune regulation and neuromuscular junction function.

### 3.2 Identification of differentially expressed genes (DEGs)

**Fig 3:**
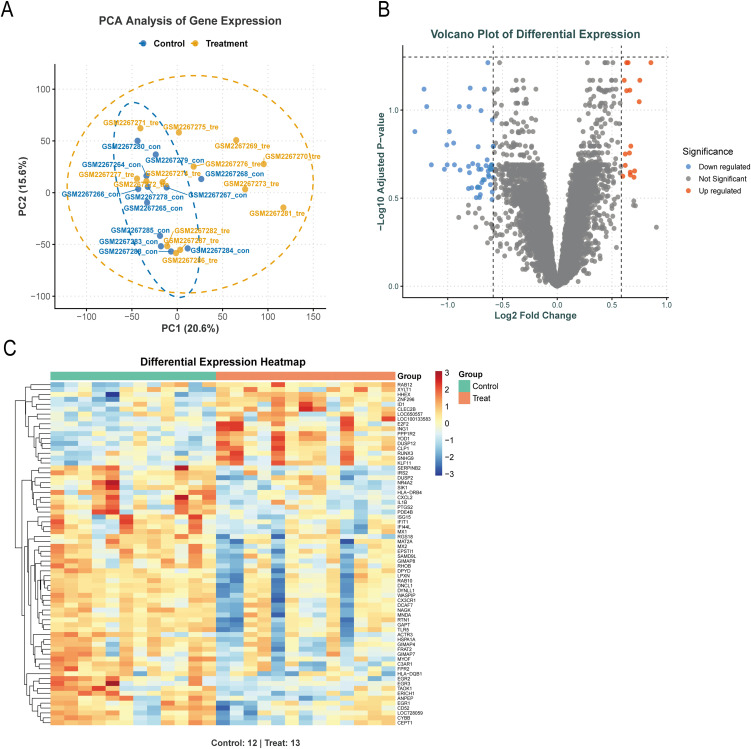
Differential gene expression analysis. (A)PCA analysis of gene expression. (B) Volcano plot of differentially expressed gene analysis of MG samples and Control samples. (C) Heat map of MG-related DEGs in the GEO Datasets.

The RNA-seq dataset (GSE85452) of MG patients was obtained from the GEO database to identify DEGs associated with MG. Principal component analysis (PCA) was performed on the gene expression profiles of the GSE85452 dataset to assess global expression patterns and sample distribution (Fig 3A). PCA showed partial separation between groups, suggesting moderate differences in global gene expression profiles. We analyzed CD14 + monocyte samples from 13 MG patients and 12 healthy controls, identifying a total of 71 DEGs (Fig 3B). The heatmap illustrates expression profiles of the 71 genes (Fig 3C), indicating significant differences in gene expression patterns between the disease group and the control group.

### 3.3 Identification of key targets through machine learning

**Fig 4:**
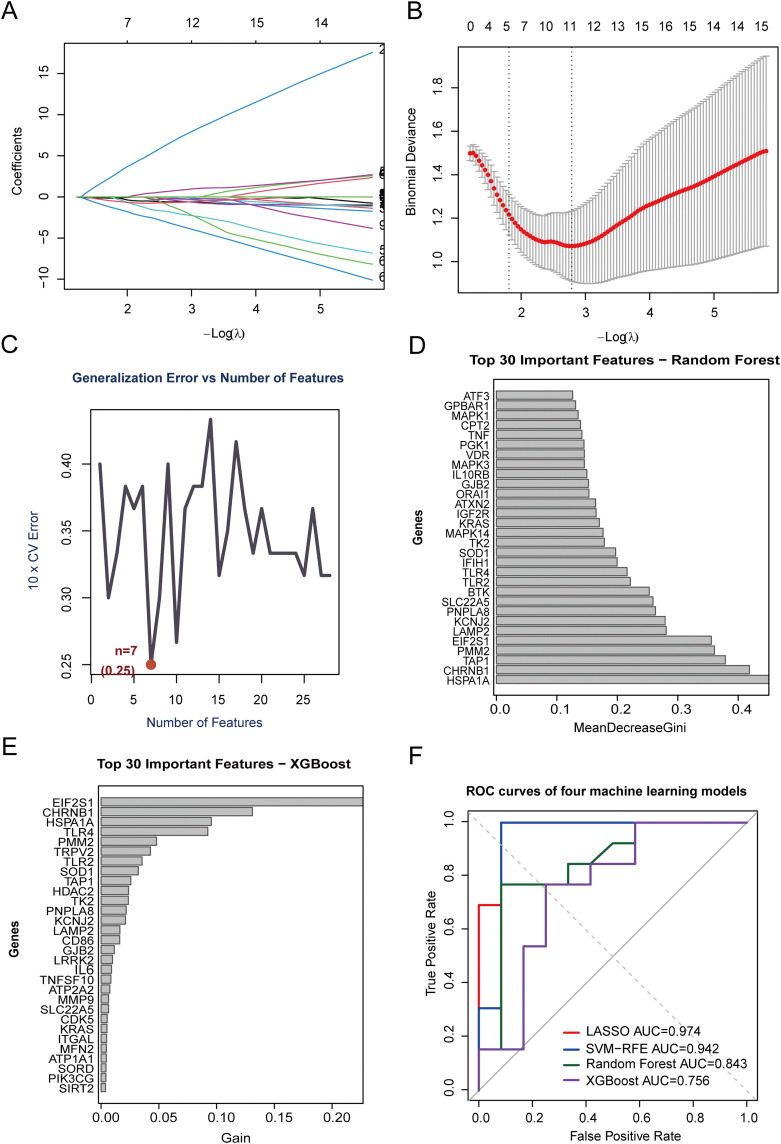
Machine learning-guided identification of core targets related to BPA-induced MG. (A) Coefficient profiles of candidate genes in the LASSO regression model.(B) Cross-validation curve for selecting the optimal penalty parameter in the LASSO model.(C) SVM-RFE cross-validation analysis showing the relationship between generalization error and the number of selected features.(D) Top 30 important features ranked by random forest based on MeanDecreaseGini.(E) Top 30 important features ranked by XGBoost.(F) ROC curves comparing the diagnostic performance of the LASSO, SVM-RFE, random forest, and XGBoost models.

To refine the candidate genes, multiple machine-learning algorithms were applied to 225 BPA-induced MG-related targets, including LASSO logistic regression, SVM-RFE, random forest, and XGBoost. In the LASSO regression model, candidate genes were screened according to the optimal penalty parameter selected by cross-validation, and 11 genes were retained as potential diagnostic targets (Fig 4A-B). SVM-RFE was further used to evaluate model performance under different feature numbers. The model achieved the lowest generalization error of 0.25 when seven genes were included, corresponding to an accuracy of 0.75 (Fig 4C). Random forest and XGBoost analyses were then performed to rank the relative importance of candidate genes, and the top 30 genes identified by each model are shown in Fig 4D and Fig 4E. Finally, ROC curve analysis was used to compare the diagnostic performance of the four machine-learning models. The results suggested that LASSO, SVM-RFE, random forest, and XGBoost all showed favorable predictive performance, supporting the reliability of the selected key targets (Fig 4F). The detailed performance metrics of the four machine-learning models are summarized in S1 Table. Based on the overlapping results and feature importance rankings, four core targets were identified for subsequent analyses: CHRNB1, KRAS, PMM2, and TLR4.

### 3.4 Validation of three key targets in MG and evaluation of their diagnostic performance

**Fig 5:**
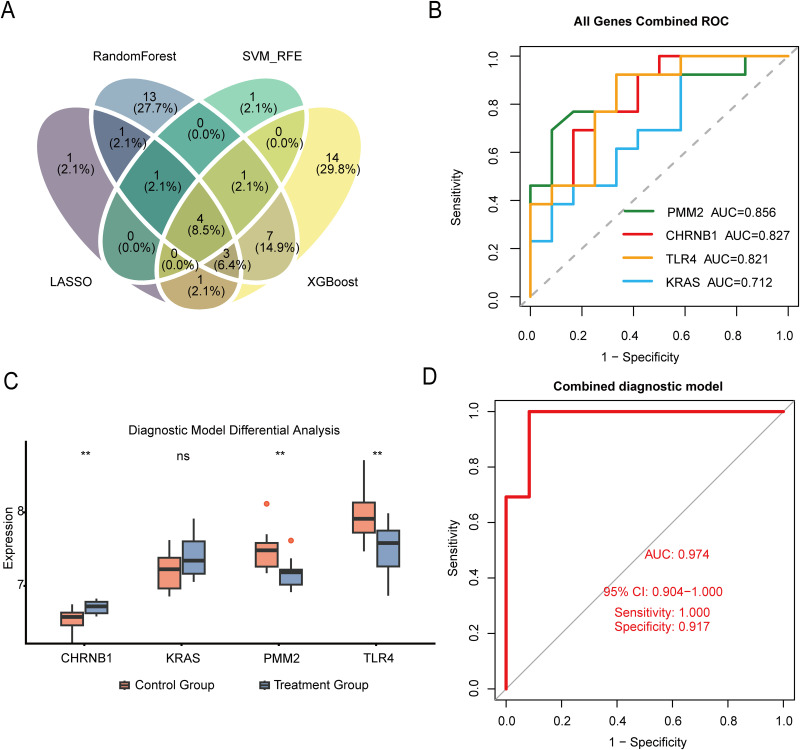
Machine-learning screening, expression validation and diagnostic evaluation of candidate genes in MG. (A) Venn diagram showing candidate genes identified by LASSO, random forest, SVM-RFE and XGBoost.(B) ROC curves of CHRNB1, KRAS, PMM2, and TLR4 based on the GSE85452 dataset. (C) Differential expression analysis of CHRNB1, KRAS, PMM2 and TLR4 between MG and control samples. ns, not significant; **P < 0.01.(D) ROC curve of the combined diagnostic model constructed using CHRNB1, PMM2, and TLR4.

The RNA-seq dataset GSE85452 was obtained from the GEO database to validate the expression patterns and diagnostic value of the candidate genes. Four machine-learning algorithms, including LASSO, random forest, SVM-RFE, and XGBoost, were used to screen candidate targets, and their overlapping genes were visualized using a Venn diagram (Fig 5A). Four candidate genes, including CHRNB1, KRAS, PMM2, and TLR4, were selected for further validation.ROC curve analysis was performed to evaluate the diagnostic performance of these candidate genes. The AUC values of CHRNB1, KRAS, PMM2, and TLR4 were 0.827, 0.712, 0.856, and 0.821, respectively (Fig 5B), indicating favorable diagnostic potential, particularly for CHRNB1, PMM2, and TLR4. Differential expression analysis showed that CHRNB1, PMM2, and TLR4 were significantly different between MG and control samples, whereas KRAS showed no significant difference (Fig 5C). Therefore, CHRNB1, PMM2, and TLR4 were selected as the three key targets for subsequent analyses.To further evaluate their combined diagnostic value, a multivariate logistic regression model was constructed based on CHRNB1, PMM2, and TLR4. ROC curve analysis showed that the combined diagnostic model achieved an AUC of 0.974, with a 95% confidence interval of 0.904–1.000, sensitivity of 1.000, and specificity of 0.917 (Fig 5D). These results suggest that the combined model based on CHRNB1, PMM2, and TLR4 has strong potential for distinguishing MG samples from control samples.

### 3.5 The correlation analysis between core genes and immune cells in MG

**Fig 6:**
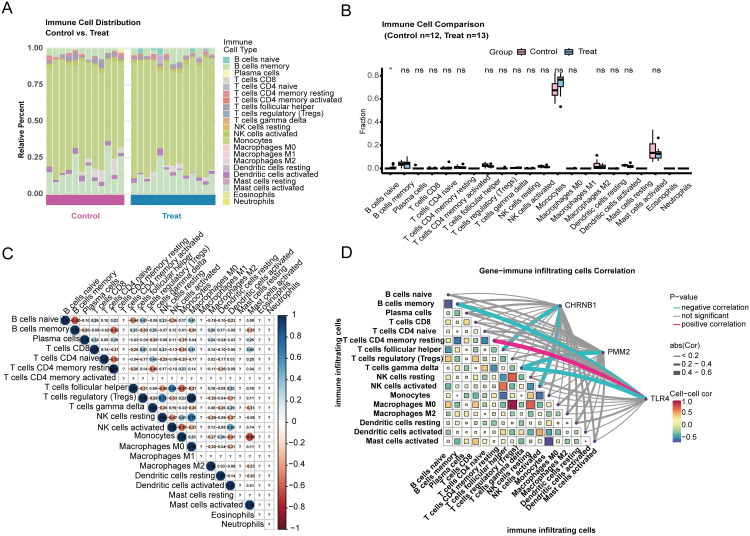
Immune microenvironment differences and gene–immune correlations in MG. (A) Heatmap showing the distribution of different immune cell types in CD14 + monocyte samples from MG patients and healthy controls. (B, C) Differential immune cell inﬁltration between MG and control groups. (D) Heatmap showing correlations between 3 model genes and 17 immune cell subsets.

A comprehensive analysis of immune cell infiltration patterns and their functional correlations between MG group and the control group was conducted. The relative proportions of immune cell subsets are shown in Fig 6A, indicating significant alterations in the MG group. The distribution of 17 immune cell populations in each sample was calculated using the CIBERSORT algorithm. Notably, naïve B cells exhibited a significantly increased infiltration score in the MG group compared with the control group MG:0.0095 ± 0.0155vs.control:0.0000 ± 0.0000,P = 0.0217 (Fig 6B).Moreover, the immune cell interaction network was significantly remodeled under disease conditions, characterized by enhanced positive correlations among key immune subsets. This pattern suggests the presence of a coordinated yet ultimately dysregulated immune response during the progression of MG (Fig 6C-D). Correlation analysis between three key model genes and 17 immune cell subsets further revealed potential immunomodulatory mechanisms. CHRNB1 showed a negative correlation with T follicular helper cells and activated NK cells, implying its potential involvement in suppressing specific pro‑inflammatory or helper immuneresponses. Both the metabolism-related gene PMM2 and the pattern-recognition receptor gene TLR4 were negatively correlated with T cells gamma delta, which may reflect the regulation of this unconventional T-cell subset by metabolic reprogramming or innate immune signaling.

### 3.6 Molecular docking and visualization of BPA with the identified MG targets

**Fig 7:**
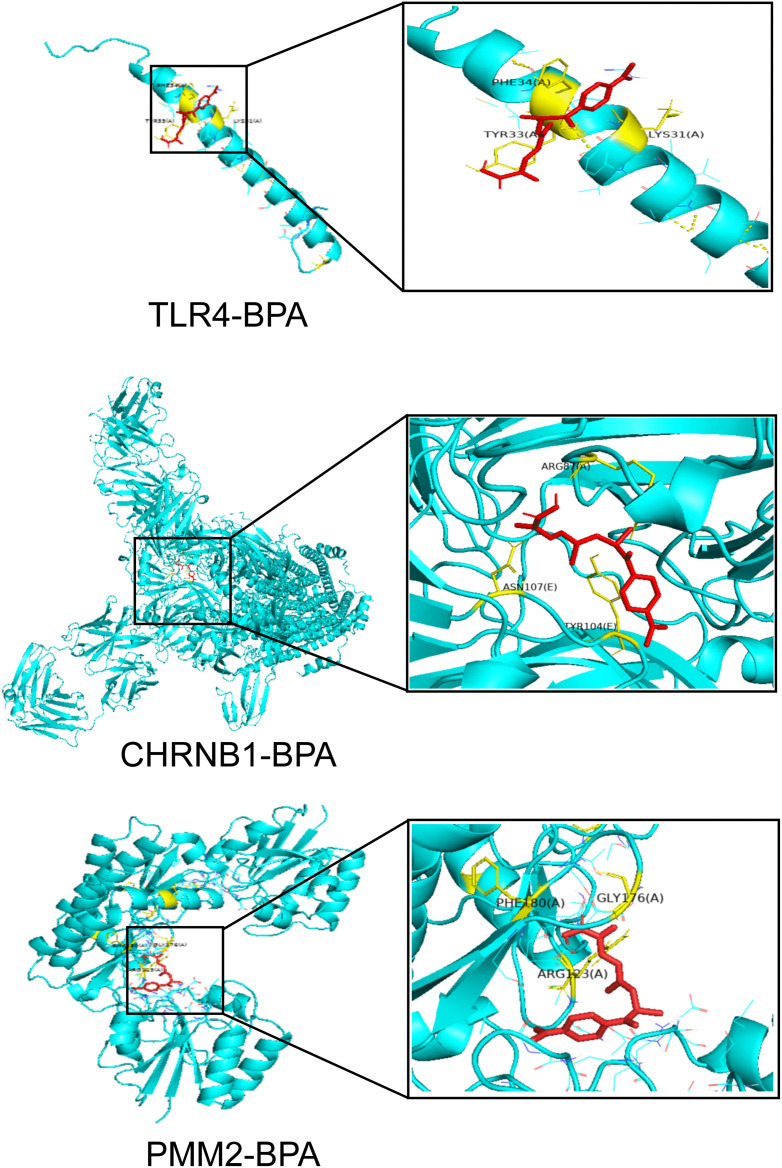
Molecular docking visualization of BPA with the identified MG targets.

To explore the intermolecular binding ability, we calculated and analyzed the binding energy of BPA with CHRNB1, PMM2 and TLR4 through molecular docking visualization (Fig 7).In general, a binding energy of less than 0 kcal·mol^-1^ means that the receptor and ligand bind spontaneously without the need for external energy, a binding energy of less than −5 kcal·mol^-1^ indicates excellent binding, and a binding energy of less than −7 kcal·mol^-1^ indicates strong binding. The binding energies of BPA with CHRNB1, PMM2, and TLR4 are −7.4 kcal/mol, −5.7 kcal/mol, and −5.8 kcal/mol, respectively, indicating that BPA has a strong binding affinity with these core targets.

### 3.7 MD simulations of BPA with the identified MG targets

**Fig 8:**
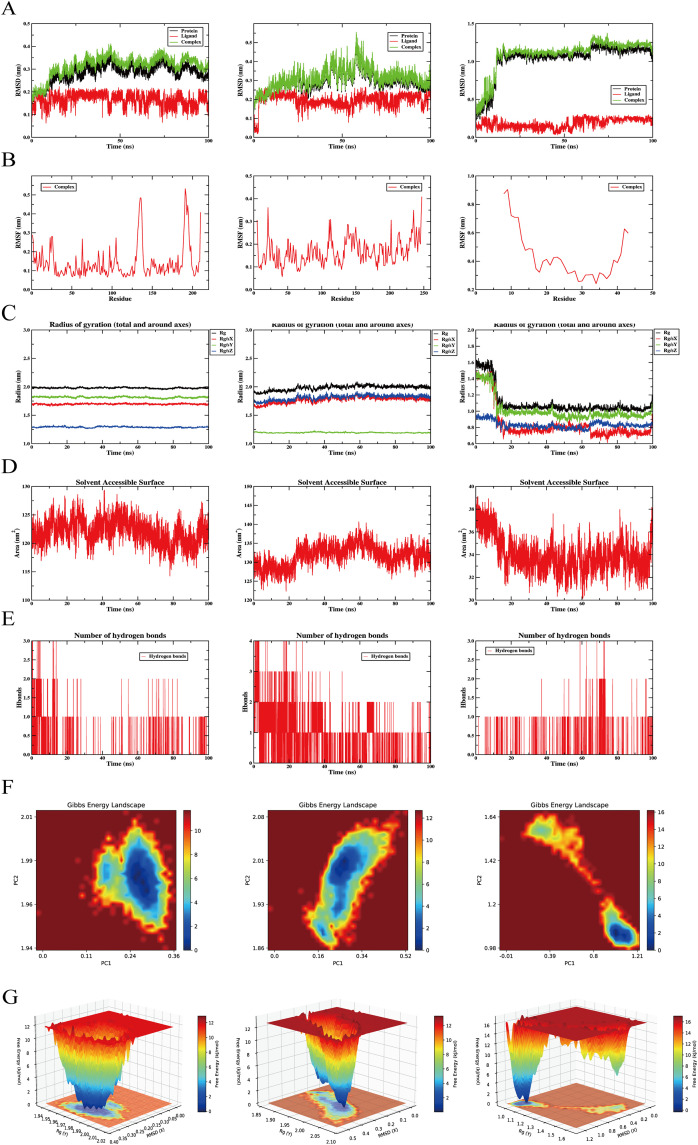
MD simulations of BPA with the identified MG targets. (A-E) The RMSD, RMSF, Rg, H-bonds Number, SASA analysis. (F-G) Free energy distribution diagram.

In this study, the binding interactions between BPA and CHRNB1, PMM2, and TLR4 were analyzed using MD simulations. RMSD analysis was used to evaluate the structural stability of the BPA-target complexes. As shown in Fig 8A, the RMSD curves of the three complexes gradually reached equilibrium during the simulation. During the equilibrated phase from 20 to 100 ns, the RMSD values of the BPA-CHRNB1, BPA-PMM2, and BPA-TLR4 complexes fluctuated within 0.255–0.413 nm, 0.227–0.556 nm, and 1.013–1.377 nm, respectively. Their mean RMSD values were 0.332 nm, 0.328 nm, and 1.176 nm, and the maximum RMSD values were 0.413 nm, 0.556 nm, and 1.377 nm, respectively. These results indicate that BPA-CHRNB1 and BPA-PMM2 exhibited relatively low RMSD fluctuations, whereas BPA-TLR4 showed a larger conformational adjustment but remained stable after equilibrium.RMSF analysis showed relatively low residue fluctuations in BPA-CHRNB1 and BPA-PMM2, while BPA-TLR4 displayed relatively higher local flexibility in some residues (Fig 8B). The smooth Rg curves further confirmed that the compactness of the complexes was maintained during the simulation (Fig 8C). Hydrogen bond analysis showed that BPA formed dynamic and transient hydrogen stability may also be maintained by other non-covalent interactions. SASA curves further reflected the overall structural stability and solvent exposure changes of the complexes during the simulation (Fig 8D-E). Conformational comparisons at five representative time points showed that BPA maintained stable binding positions within the protein binding pockets. Free energy landscape analysis revealed dominant minimum energy regions, indicating stable interactions between BPA and the identified MG-related targets (Fig 8F-G).To further evaluate the thermodynamic stability of the BPA-target protein complexes, MM-PBSA-based binding free energy analysis was performed based on the equilibrated MD trajectories. The calculated binding free energies of BPA-CHRNB1, BPA-PMM2, and BPA-TLR4 were −125.055 kJ/mol, −124.070 kJ/mol, and −132.307 kJ/mol, respectively, corresponding to −29.889 kcal/mol., −29.653 kcal/mol, and −31.622 kcal/mol The negative binding free energy values indicated that BPA could form thermodynamically favorable interactions with all three target proteins. Among them, BPA-TLR4 showed the lowest binding free energy, suggesting relatively stronger binding stability. Detailed energy decomposition results are provided in S2 Table.

### 3.8 Effects of BPA on cell viability and related gene expression

**Fig 9:**
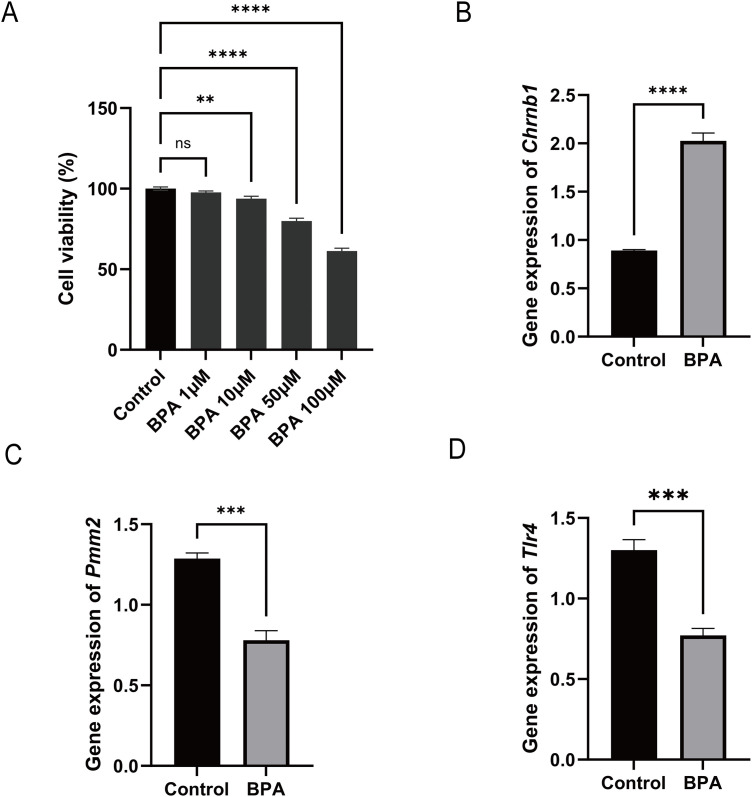
Effects of BPA on cell viability and related gene expression. (A) Changes in cell viability after treatment with different concentrations of BPA. Compared with the control group, the 1μM BPA-treated group showed no significant difference in cell viability, whereas the 10μM, 50μM, and 100μM BPA-treated groups showed significantly reduced cell viability. (B) The expression level of Chrnb1 was significantly increased after treatment with 50μM BPA. (C) The expression level of Pmm2 was significantly decreased after treatment with 50 μM BPA. (D) The expression level of Tlr4 was significantly decreased after treatment with 50μM BPA.Data are presented as the mean ± SEM. One-way ANOVA was used for panel A, and comparisons between two groups were used for panels B–D. ns indicates no statistically significant difference; **P < 0.01, ***P < 0.001, and ****P < 0.0001.

To evaluate the effect of BPA on cell viability, cells were treated with different concentrations of BPA, followed by measurement of cell viability. As shown in Fig 9A, compared with the control group, treatment with 1μM BPA did not significantly affect cell viability. However, cell viability was significantly reduced after treatment with 10μM BPA. When the BPA concentration was increased to 50μM and 100μM, cell viability decreased further, with highly significant differences. In particular, cell viability in the 50μM and 100 μM BPA-treated groups was markedly lower than that in the control group, suggesting that BPA inhibits cell viability in a dose-dependent manner. Based on the results of the cell viability assay, 50μM BPA was selected for subsequent analysis of related gene expression. The qRT-PCR results showed that, compared with the control group, treatment with 50 μM BPA significantly upregulated the mRNA expression level of Chrm1 (Fig 9B). Meanwhile, the mRNA expression level of Pmm2 was significantly decreased (Fig 9C), whereas that of Tlr4 was significantly decreased (Fig 9D).

### 3.9 Effects of BPA on the protein expression of related genes

**Fig 10:**
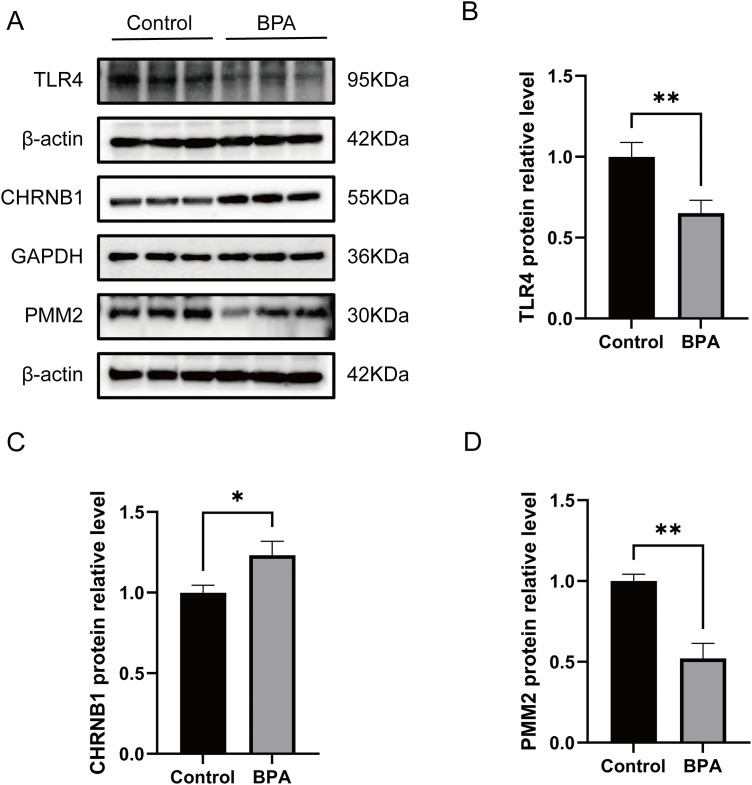
Effects of BPA on the protein expression of related genes. (A) Representative Western blot bands of TLR4, CHRNB1, and PMM2 in the control and BPA-treated groups. β-actin or GAPDH was used as the internal reference. (B) Quantitative analysis of TLR4 protein expression. (C) Quantitative analysis of CHRNB1 protein expression. (D) Quantitative analysis of PMM2 protein expression. Data are presented as mean ± SD. *P < 0.05, **P < 0.01.

To further validate the regulatory effects of BPA on key candidate genes, Western blot analysis was performed to detect the protein expression levels of TLR4, CHRNB1, and PMM2 after BPA treatment(Fig 10A). Uncropped and unprocessed Western blot images provided in S1 File. Quantitative analysis showed that, compared with the control group, BPA treatment significantly decreased the protein expression level of TLR4 (Fig 10B). In contrast, the protein expression level of CHRNB1 was significantly increased after BPA exposure (Fig 10C). Meanwhile, PMM2 protein expression was markedly reduced in the BPA-treated group compared with the control group (Fig 10D). These findings indicate that BPA exposure can alter the expression of disease-related proteins, including the downregulation of TLR4 and PMM2 and the upregulation of CHRNB1.

## 4 Discussion

The traditional view holds that MG is closely related to genetics and thymus abnormalities [26]. However, genetic factors alone cannot fully explain the increasing incidence and regional differences of MG, suggesting that gene–environment interactions may also contribute to disease development. Environmental pollutants may act as exogenous triggers that disturb immune tolerance in susceptible individuals. BPA is a widely distributed environmental endocrine disruptor found in plastic products, and human biomonitoring studies have shown that BPA is commonly detected in the body through dietary, dermal, and other exposure routes [27]. In addition to its endocrine-disrupting effects, BPA has been reported to regulate immune and inflammatory responses, which may overlap with mechanisms involved in autoimmune diseases.

In this study, 225 overlapping targets between BPA-related toxicological targets and MG-related targets were identified through network toxicology analysis. Functional enrichment analysis suggested that these targets were mainly involved in ion transport, membrane potential regulation, muscle-related biological processes, inflammatory signaling, apoptosis, endocrine-related pathways, and AGE-RAGE signaling. These biological processes and pathways are closely associated with immune regulation, chronic inflammation, and neuromuscular function [28–31].Previous studies have also suggested that the RAGE-S100B axis may contribute to immune activation and disease progression in experimental autoimmune MG, and altered RAGE-related signaling has been reported in patients with MG [32–34].These findings indicate that BPA may be associated with MG-related immune and neuromuscular dysfunction through multiple biological pathways.

Differential expression analysis based on the GSE85452 dataset identified 71 DEGs between MG patients and healthy controls, indicating distinct transcriptomic alterations in CD14 + monocyte samples. Machine-learning analysis further screened four candidate genes, including CHRNB1, KRAS, PMM2, and TLR4. Among them, CHRNB1, PMM2, and TLR4 were significantly differentially expressed between MG and control samples, whereas KRAS showed no significant difference. ROC analysis showed that CHRNB1, PMM2, and TLR4 had favorable diagnostic performance, with AUC values of 0.827, 0.856, and 0.821, respectively. These findings suggest that CHRNB1, PMM2, and TLR4 may be closely associated with MG-related molecular alterations and may serve as potential candidate biomarkers.

Immune infiltration analysis revealed that the local immune microenvironment of MG was reshaped, and the core targets showed significant associations with the infiltration levels of specific immune cell subsets, such as follicular helper T cells, activated NK cells, and γδ T cells.These results suggest that the identified key targets may be involved in immune microenvironment remodeling in MG. However, these correlations do not establish direct causality and should be further validated by functional experiments.

To verify the potential interaction between BPA and the core targets at the structural level, molecular docking and MD simulations were performed. Preliminary results indicated that BPA could bind to CHRNB1, PMM2, and TLR4, with binding energies of −7.4, −5.7, and −5.8 kcal/mol, respectively.MD simulations further suggested that the BPA-CHRNB1 and BPA-PMM2 complexes exhibited relatively stable RMSD fluctuations, whereas the BPA-TLR4 complex showed larger conformational adjustment but remained stable after equilibrium.

Together, these computational results provide structural evidence supporting the possible interaction between BPA and CHRNB1, PMM2, and TLR4. Nevertheless, molecular docking and MD simulations can only suggest potential binding stability, and further experiments are required to determine whether BPA directly affects the biological functions of these proteins.

According to the methodological guidance for network pharmacology evaluation, biological experimental validation is recommended to improve the reliability of computational predictions [35]. Therefore, we performed in vitro validation using C2C12 cells. BPA treatment reduced C2C12 cell viability in a dose-dependent manner, and 50 μM BPA was selected for subsequent molecular validation. The qRT-PCR and Western blot results showed that 50 μM BPA increased Chrnb1 mRNA and CHRNB1 protein levels, while decreasing Pmm2 and Tlr4 mRNA levels as well as PMM2 and TLR4 protein levels. These expression trends were broadly consistent with the transcriptomic validation results in peripheral blood-derived CD14 + monocyte samples from MG patients and healthy controls, in which CHRNB1 was upregulated whereas PMM2 and TLR4 were downregulated. These findings provide preliminary experimental support that BPA exposure may induce molecular changes related to neuromuscular signaling, immune regulation, and metabolic processes in muscle cells. However, because the validation was performed in mouse C2C12 myoblasts, the results should be interpreted as supportive experimental evidence rather than complete replication of the human MG transcriptomic profile.

Based on the docking and MD simulation results, BPA may affect MG-related biological processes at several mechanistic levels. First, BPA may interact with CHRNB1, PMM2, and TLR4 through non-covalent interactions, which may induce local conformational perturbations or affect the stability of protein functional domains. Such conformational changes may further influence protein function. CHRNB1 encodes the β1 subunit of the nicotinic acetylcholine receptor, which is essential for acetylcholine receptor assembly and neuromuscular junction signal transmission [36]. Mutations in this gene are closely related to congenital myasthenic syndrome (CMS), especially fast-channel CMS [37]. The predicted interaction between BPA and CHRNB1 suggests that BPA may potentially interfere with neuromuscular receptor-related processes, although this requires further functional validation.

PMM2 is a key enzyme in the N-linked glycosylation pathway [38], and loss-of-function mutations in PMM2 are the major cause of PMM2-CDG [39]. This multisystem disease affects the nervous system, liver, endocrine system, and coagulation system and may present with developmental delay, cerebellar malformations, and coagulation disorders [40]. Our simulation results suggested that BPA may interact with PMM2. Given the known endocrine- and metabolism-disrupting properties of BPA, this interaction raises the possibility that BPA may be associated with PMM2-related enzymatic activity and protein glycosylation processes. However, further enzymatic activity assays are needed to verify this hypothesis.

Toll-like receptors are key pattern-recognition receptors that play important roles in innate immunity [41]. They are mainly expressed in innate immune cells such as dendritic cells, macrophages, and neutrophils, as well as in some epithelial cells, and serve as important sensors of host defense [42]. TLR4 is a key innate immune receptor that recognizes pathogen-associated or damage-associated molecular patterns and activates inflammatory signaling pathways, including NF-κB-mediated responses [43–45]. Previous studies have reported that BPA can regulate TLR4-related signaling and affect inflammatory responses [15,46]. In the present study, TLR4 was significantly downregulated in peripheral blood-derived CD14 + monocyte samples from MG patients, and Tlr4 mRNA and TLR4 protein levels were also decreased in C2C12 cells after 50 μM BPA treatment. This consistent downregulation suggests that TLR4 may be involved in BPA-related immune regulation and MG-associated molecular alterations. However, the present study only validated the expression changes of candidate targets at the mRNA and protein levels, while receptor activity, downstream signaling activation, and inflammatory cytokine release were not examined. Therefore, further experiments are needed to determine whether BPA directly affects TLR4 signaling activity and inflammatory cytokine production.

Taken together, our findings suggest that BPA exposure may be linked to MG-related biological alterations through several interconnected processes, including ion transport and membrane potential regulation, reduced muscle cell viability, immune-related pathway changes, and altered expression of neuromuscular and metabolic genes. Notably, the increased expression of Chrnb1 and the decreased expression of Pmm2 and Tlr4 observed after BPA treatment were generally consistent with the expression patterns identified in peripheral blood-derived CD14 + monocyte samples. These results support the possibility that BPA may be associated with molecular changes in muscle cells related to neuromuscular signaling, immune regulation, and metabolic function. However, given the differences between the C2C12 cell model and the human MG pathological context, these in vitro findings should be regarded as supportive evidence rather than direct replication of clinical MG conditions.

Compared with previous studies, most existing evidence has mainly focused on the endocrine-disrupting, metabolic, and inflammatory effects of BPA, whereas direct studies investigating the association between BPA exposure and MG remain limited. Previous reports have suggested that BPA may regulate immune and inflammatory responses through TLR4-related signaling, oxidative stress, and cytokine production. These mechanisms are partially consistent with the immune dysregulation and inflammatory activation observed in MG. However, the specific molecular links between BPA exposure and MG-related targets have not been fully clarified.

The novelty of the present study lies in the integration of network toxicology, machine learning, transcriptomic validation, immune infiltration analysis, molecular docking, molecular dynamics simulation, and in vitro qRT-PCR and Western blot validation to systematically explore the potential relationship between BPA exposure and MG. Through this multi-level integrative framework, we identified CHRNB1, PMM2, and TLR4 as potential molecular links connecting BPA exposure with immune imbalance and neuromuscular dysfunction. In vitro validation further showed that 50 μM BPA reduced C2C12 cell viability, increased Chrnb1 mRNA and CHRNB1 protein levels, and decreased Pmm2 and Tlr4 mRNA levels as well as PMM2 and TLR4 protein levels.

Overall, this study provides computational and preliminary experimental evidence suggesting that BPA exposure may be associated with MG-related molecular and immune dysregulation. By linking BPA exposure to core molecular targets, immune infiltration changes, and neuromuscular junction-related dysfunction, our findings provide a theoretical basis for future experimental validation and environmental risk assessment.

Nevertheless, these findings should be interpreted with caution. First, this study was mainly based on computational prediction, publicly available transcriptomic datasets, and preliminary in vitro validation; therefore, the results do not establish a direct causal relationship between BPA exposure and MG. Second, the GSE85452 dataset was derived from peripheral blood-derived CD14 + monocyte samples, whereas the in vitro validation was performed in mouse C2C12 myoblasts. Although C2C12 cells have been used in previous MG-related studies as a skeletal muscle cell model, differences in species, sample type, and biological context should still be taken into account when interpreting the validation results obtained from this cell model. Third, the present study validated the expression changes of candidate genes at both mRNA and protein levels, receptor activity, inflammatory cytokine production, and neuromuscular junction-related functional changes were not examined. Finally, in vivo experiments and clinical studies incorporating BPA exposure data are needed to further confirm whether BPA contributes to MG onset or progression.

## 5 Conclusion

In summary, this study suggests that BPA exposure may be associated with MG-related molecular alterations through the regulation of ion transport, neuromuscular signaling, immune regulation, and metabolic pathways. Network toxicology, machine-learning analysis, transcriptomic validation, molecular docking, and molecular dynamics simulations identified CHRNB1, PMM2, and TLR4 as potential key targets. In vitro validation further showed that 50 μM BPA reduced C2C12 cell viability, increased Chrnb1 mRNA and CHRNB1 protein levels, and decreased Pmm2 and Tlr4 mRNA levels as well as PMM2 and TLR4 protein levels. These findings suggest that BPA may be involved in muscle cell injury and immune-related molecular alterations, providing preliminary clues for understanding the potential role of environmental exposure in MG-related pathogenesis. Further in vivo experiments and clinical studies are required to confirm these findings.

## Supporting information

S1 Table The detailed performance metrics of the four machine-learning models. (CSV)

S2 Table The detailed energy decomposition results. (XLTX)

S1 File Uncropped and unprocessed Western blot images. (PDF)

## Abbreviations

MG: myasthenia gravis
AChR: acetylcholine receptor
LRP4: lipoprotein receptor-related protein 4
BPA: bisphenol A
EDCs: endocrine-disrupting chemicals
MD: molecular dynamics
GO: gene ontology
BP: biological process
CC: cellular component
MF: molecular function
LASSO: least absolute shrinkage and selection operator
SVM: support vector machine
RFE: recursive feature elimination
AUC: area under the curve
ROC: receiver operating characteristic
CHRNB1: cholinergic receptor nicotinic beta 1 subunit
PMM2: phosphomannomutase 2
TLR4: toll-like receptor 4
