# Supplementary material for: Bisphenol A exposure in myasthenia gravis: Potential targets and mechanisms revealed by network toxicology and molecular dynamics
Source: PLoS One. 2026 Jul 28;21(7):e0354138. doi: 10.1371/journal.pone.0354138 (PMC13412062; doi:10.1371/journal.pone.0354138)
Supplement: S1 File — (PDF) [file pone.0354138.s003.pdf]

The corresponding original uncropped and minimally adjusted blot images have been provided as Supporting Information file “S1 Raw images”, with figure/panel labels, molecular weight markers, lane information, and unused lanes clearly indicated.

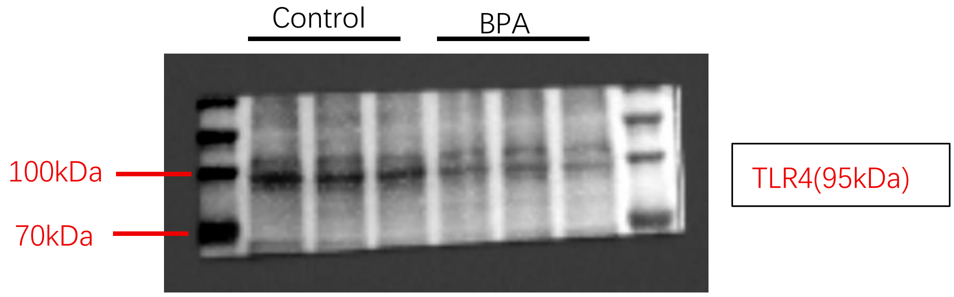

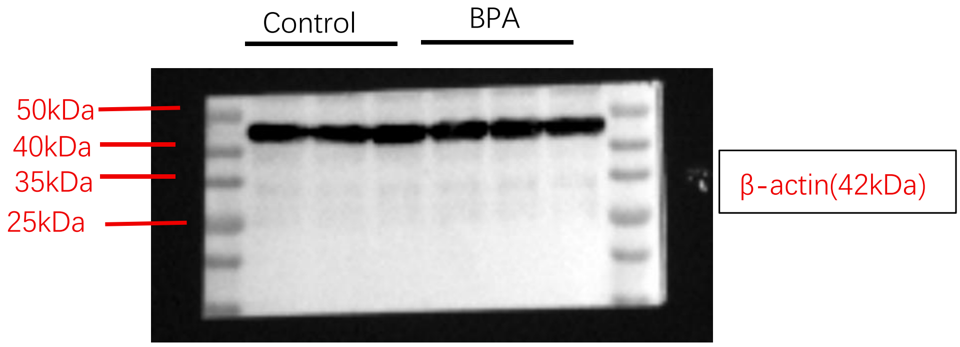

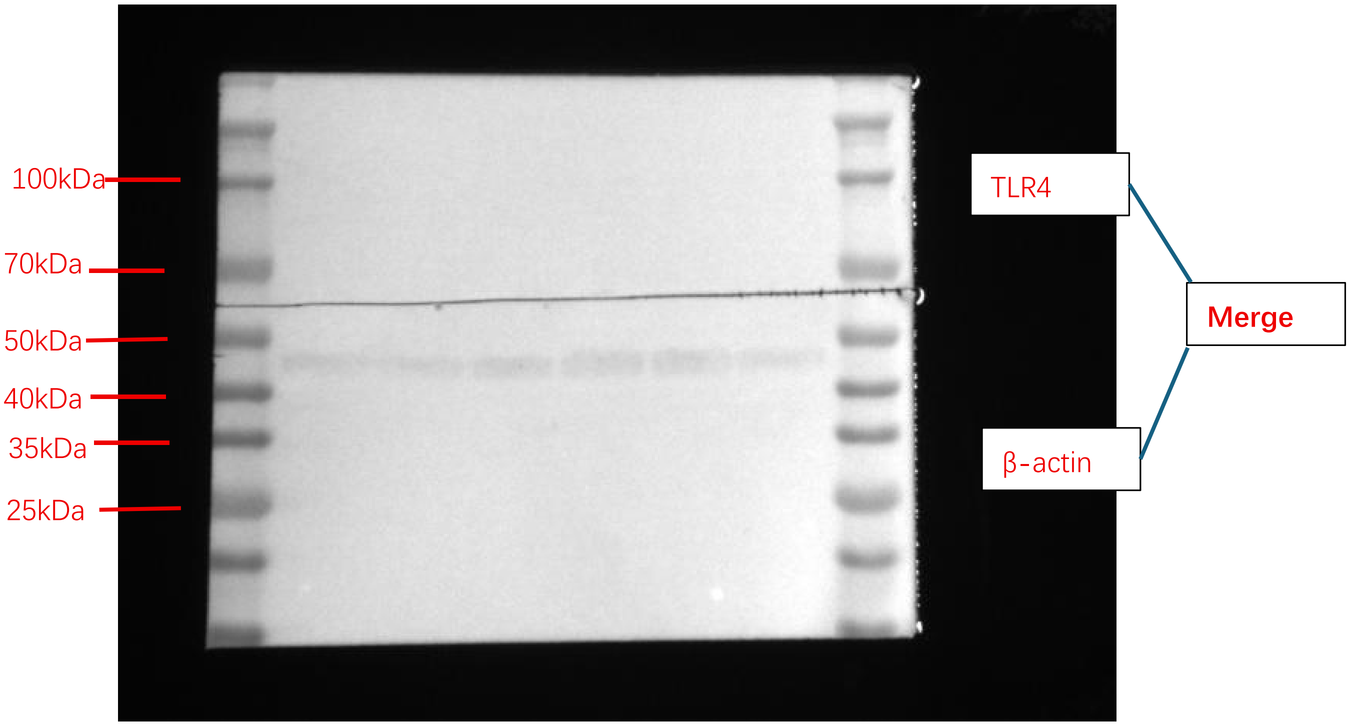

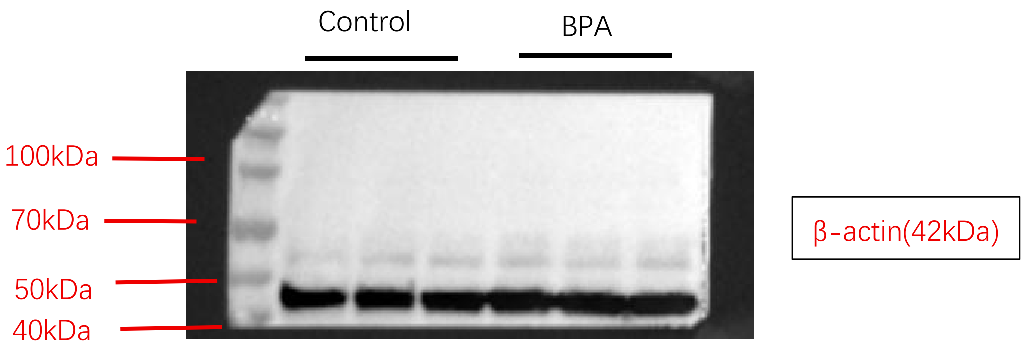
Control
BPA
100kDa
70kDa
50kDa
40kDa
β-actin(42kDa)

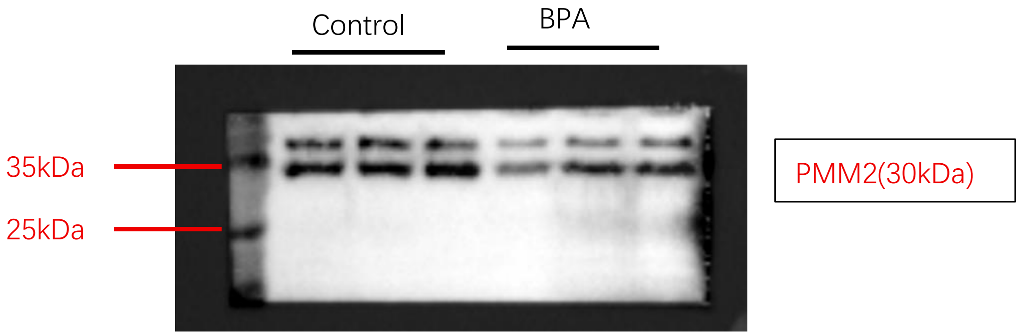
Control
BPA
35kDa
25kDa
PMM2(30kDa)

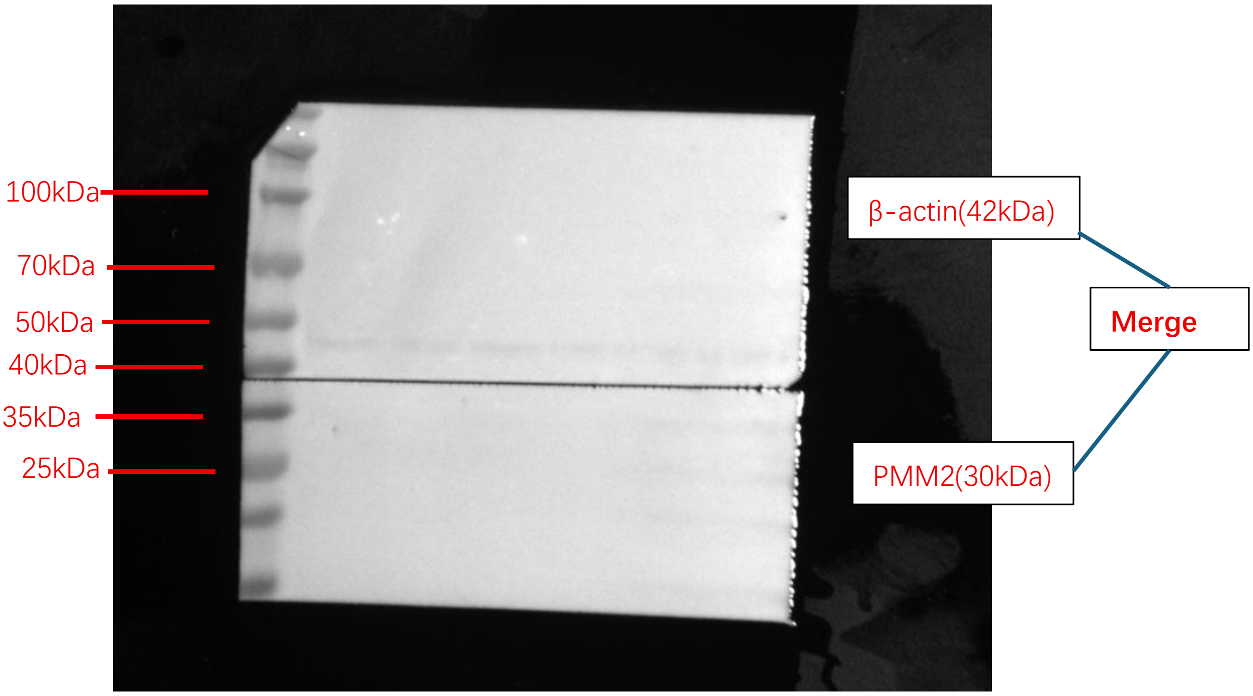
100kDa
70kDa
50kDa
40kDa
35kDa
25kDa
β-actin(42kDa)
Merge
PMM2(30kDa)

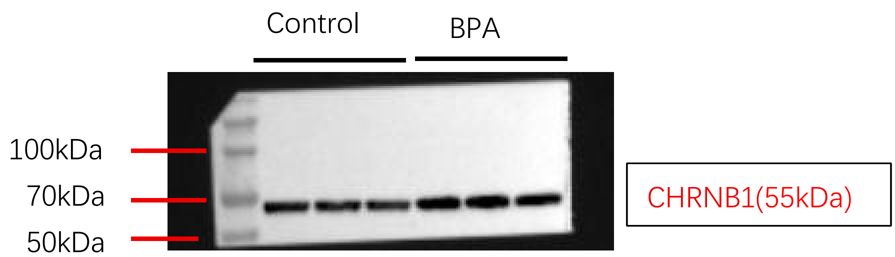
Control
BPA
100kDa
70kDa
50kDa
CHRNB1(55kDa)

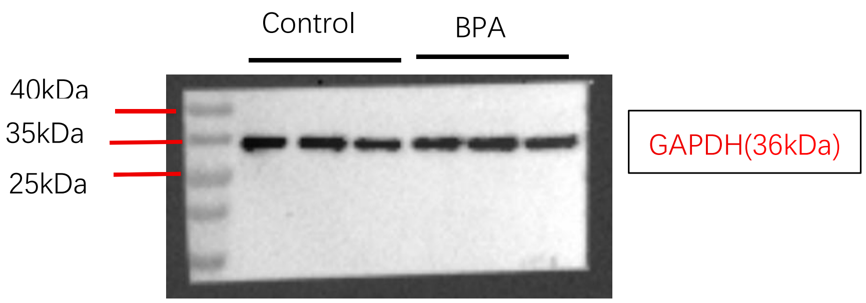
Control
BPA
40kDa
35kDa
25kDa
GAPDH(36kDa)

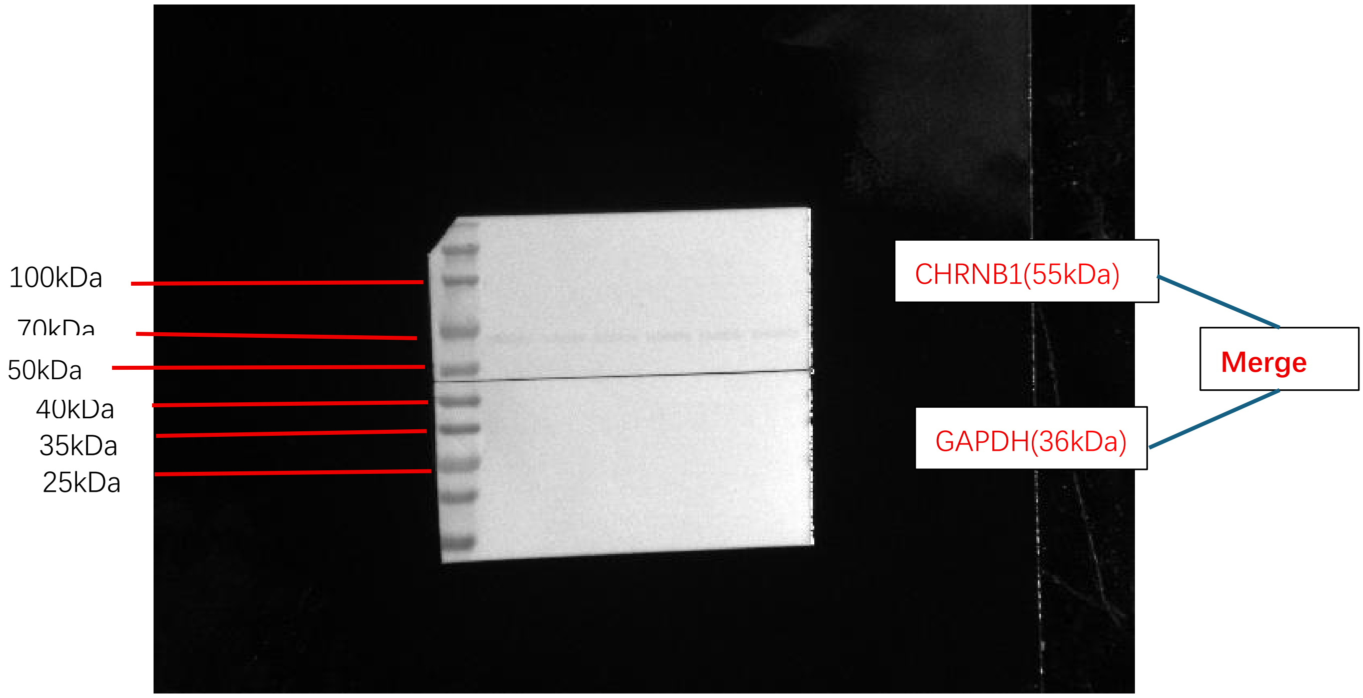
100kDa
70kDa
50kDa
40kDa
35kDa
25kDa
CHRNB1(55kDa)
Merge
GAPDH(36kDa)
